# Electron Tomography Analysis of Tick-Borne Encephalitis Virus Infection in Human Neurons

**DOI:** 10.1038/srep10745

**Published:** 2015-06-15

**Authors:** Tomáš Bílý, Martin Palus, Luděk Eyer, Jana Elsterová, Marie Vancová, Daniel Růžek

**Affiliations:** 1 Institute of Parasitology, Biology Centre of the Academy of Sciences of the Czech Republic, Branišovská 31, CZ-37005 České Budějovice, Czech Republic; 2 Faculty of Science, University of South Bohemia, Branišovská 31, CZ-37005 České Budějovice, Czech Republic; 3 Department of Virology, Veterinary Research Institute, Hudcova 70, CZ-62100 Brno, Czech Republic

## Abstract

Tick-borne encephalitis virus (TBEV) causes serious, potentially fatal neurological infections that affect humans in endemic regions of Europe and Asia. Neurons are the primary target for TBEV infection in the central nervous system. However, knowledge about this viral infection and virus-induced neuronal injury is fragmental. Here, we directly examined the pathology that occurs after TBEV infection in human primary neurons. We exploited the advantages of advanced high-pressure freezing and freeze-substitution techniques to achieve optimal preservation of infected cell architecture. Electron tomographic (ET) reconstructions elucidated high-resolution 3D images of the proliferating endoplasmic reticulum, and individual tubule-like structures of different diameters in the endoplasmic reticulum cisternae of single cells. ET revealed direct connections between the tubule-like structures and viral particles in the endoplasmic reticulum. Furthermore, ET showed connections between cellular microtubules and vacuoles that harbored the TBEV virions in neuronal extensions. This study was the first to characterize the 3D topographical organization of membranous whorls and autophagic vacuoles in TBEV-infected human neurons. The functional importance of autophagy during TBEV replication was studied in human neuroblastoma cells; stimulation of autophagy resulted in significantly increased dose-dependent TBEV production, whereas the inhibition of autophagy showed a profound, dose-dependent decrease of the yield of infectious virus.

Tick-borne encephalitis virus (TBEV), a member of the *Flaviviridae* family, genus *Flavivirus*, causes tick-borne encephalitis (TBE) in humans, a neuroinfection prevalent in large areas of Europe and North-eastern Asia. Humans develop a febrile illness, and a subset of cases progress to neurological manifestations ranging from mild meningitis to severe encephalomyelitis^1^,^2^. Despite the medical importance of TBE, some crucial steps in the development of encephalitis remain poorly understood. TBEV is mainly transmitted to the host when infected ticks feed. Virus replication is first detected in draining lymph nodes; this is followed by development of viremia; during the secondary viremic phase, the virus crosses the blood-brain barrier (BBB) and enters the brain^3^. Major hallmarks of TBEV neuropathogenesis are neuroinflammation, followed by neuronal death^4^,^5^, and disruption of the BBB^3^,^6^. Neuronal injury may be directly caused by viral infection, but destruction has also been attributed to infiltrating immunocompetent cells (mainly CD8^+^ T-cells), inflammatory cytokines, and activated microglial cells^2^,^7^,^8^. Tissue culture models of TBEV infections in primary neurons can distinguish between injuries caused by the virus and those caused by the immune response^9^,^10^. Various cellular models, including neuronal cell lines, primary cultures of embryonic or neonatal mouse and rat neuronal cells, and neurons derived from embryonic stem cells, have been used to explore infections with various neurotropic viruses (e.g., polio, herpes simplex type 1, varicella-zoster, West Nile, Japanese encephalitis, and rabies)^10^,^11^,^12^,^13^. We previously examined TBEV infections in human neural (neuroblastoma, glioblastoma, and medulloblastoma) cell lines^14^. On the ultrastructural level, the infection caused massive morphological changes in cells, including the proliferation and rearrangement of rough endoplasmic reticulum (RER) and signs of apoptosis or necrosis^14^.

Recently, replication features of neurotropic flaviviruses, West Nile virus, Japanese encephalitis virus, and TBEV were compared in primary mouse neuronal cultures. Viral antigen accumulation in neuronal dendrites was induced to a greater extent in a TBEV infection than in infections with the other flaviviruses^15^. TBEV replication induced characteristic ultrastructural membrane alterations in neurites, known as laminal membrane structures (LMSs)^15^. However, conventional chemical fixation for sample visualization presents obstacles in obtaining sufficient morphological detail^16^. Therefore, in the present study, we exploited the advantages of high-pressure freezing and freeze-substitution techniques to improve the preservation of virally modified structures in TBEV-infected neurons.

Here, we visualized TBEV infections in primary human neurons in three-dimensional (3D) space at ultrastructural resolution with electron tomography. To the best of our knowledge, this study was the first to visualize the architecture of cellular components involved in TBEV replication and transport in neurons and the major ultrastructural changes that occur in response to TBEV infections. These novel data revealed the neuronal injury caused by TBEV infections, independent of the immune system response. Our results may facilitate the development of novel strategies for treating this serious human neuroinfection.

## Results

### Replication of TBEV and distribution of virus antigen in human neurons

**Figure 1:**
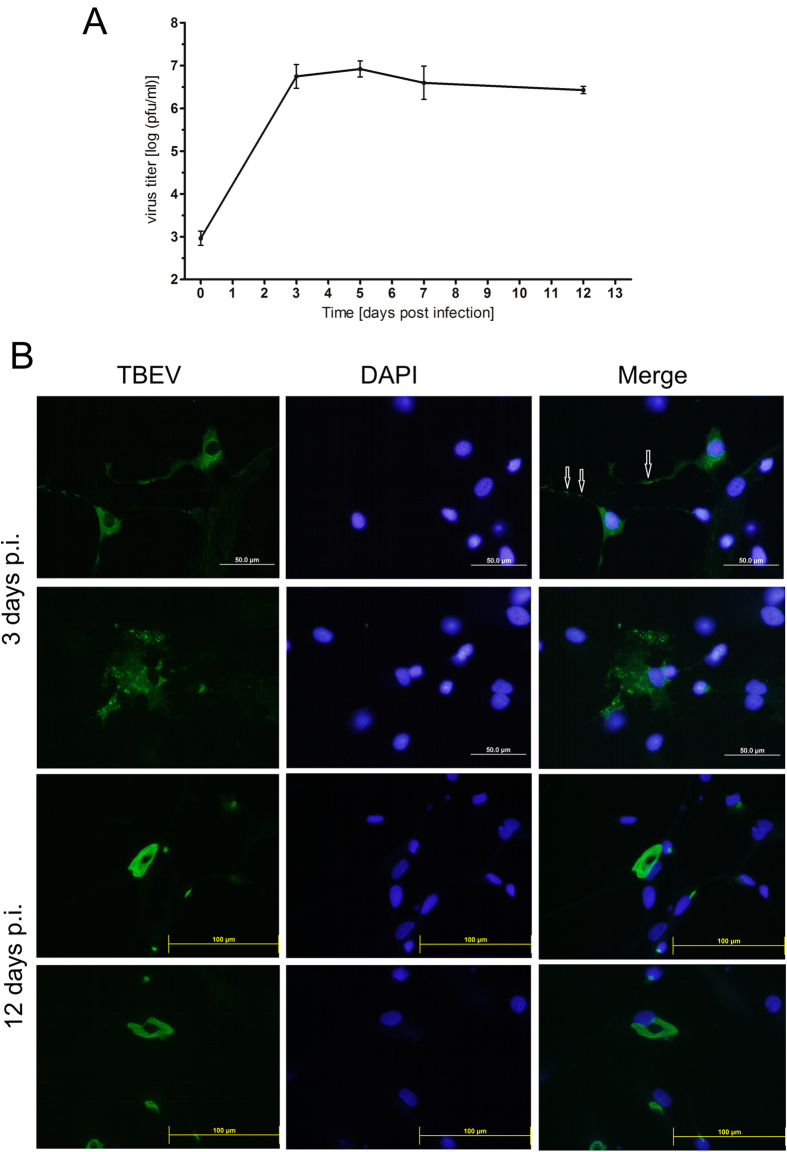
TBEV can infect human neurons. (**A**) TBEV titers in culture supernatants from HNs collected at 0, 3, 5, 7, and 12 days post-infection (p.i.) were determined in plaque assays with porcine kidney stable cells. Viral titers are expressed as pfu/ml. Data represent means ± SEM. (**B**) HNs grown and fixed on slides at days 3 and 12 p.i. were stained with anti-flavivirus envelope antibody (green) and counterstained with DAPI (blue). TBEV-infected HNs immunostained with flavivirus-specific antibody demonstrated virus replication in the cytoplasm at an early time point (3 days p.i.); antigen accumulated into aggregates at later a time point (12 days p.i.). Mock-infected HNs stained with primary and secondary antibodies were used as a negative control, and did not exhibit any TBEV antigen staining (not shown). The arrows indicate accumulation of viral antigen in dendrites of the infected HNs.

We employed a plaque assay to determine TBEV infection and replication kinetics in human neurons (HNs; Fig. 1A). The HNs were infected with TBEV, and at 0, 3, 5, 7, and 12 days post infection (p.i.), cell supernatants were collected. Productive TBEV replication was detected in the form of released virions on day 3 p.i. The virus titer in the culture supernatant remained the same until the end of the experiment (Fig. 1A).

**Figure 2:**
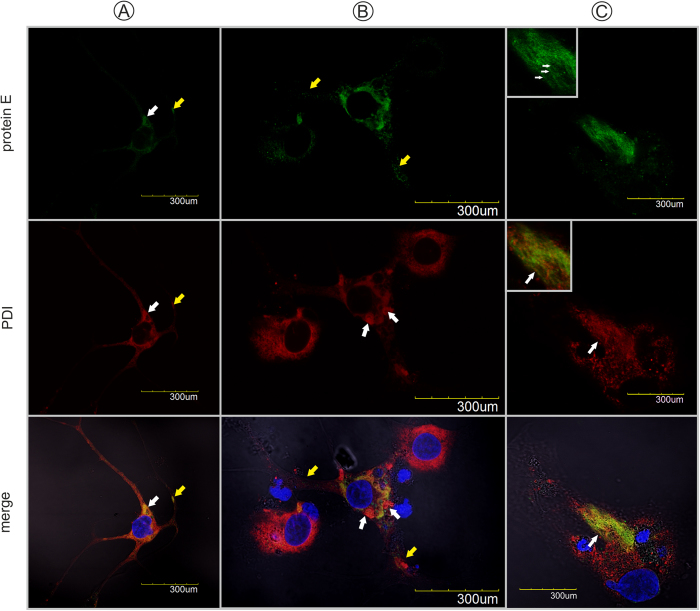
Confocal images of human neurons infected with TBEV. Neurons were fixed at (**A**,**B**) 3 days p.i. and (**C**) 12 days p.i. and double immunolabeled with antibodies against TBEV protein E (green) (*top panels*), PDIA3 (red) (*middle panels*), and (*merge panels*) counterstained with DAPI. (**A**) Replication complexes were observed in the perikaryon (white arrow) and dendrites (yellow arrow). (**B**) Infected cells with intact endoplasmic reticulum networks were observed next to infected cells with large whorls (white arrows). (**C**) Localization of viral protein E in the numerous longitudinal fibers (white arrows) associated with the ER.

Immunofluorescence staining was used to assess viral antigen distribution in HNs. Viral antigen was not detected in mock-infected HNs. Immunofluorescence staining revealed that TBEV antigen was mostly distributed diffusely throughout the entire neuron bodies at early time points after infection (Fig. 1B). However, at later time points, we observed brightly stained aggregates of viral antigen in some cells (Fig. 1B). A co-localization study with protein disulphide isomerase family a, member 3 (PDIA3) antigen (also known as Erp57, Er-60, and GRP58) suggested that the viral antigen was localized primarily in the hypertrophied, rearranged RER. Occasionally, we observed viral antigen accumulation in the dendrites of TBEV-infected HNs (Figs. 1B,2A,B, yellow arrows). Viral antigen also accumulated in association with RER alterations, such as large whorl formations (Fig. 2B, white arrows), or in places that exhibited a local loss of network structure (Fig 2C). In the latter locations, RER alterations were accompanied by the presence of numerous longitudinal fibers (white arrows) that were positively immunolabeled with anti-viral protein E (Figs. 1B,2C).

### TBEV-induced RER alterations and formation of tubule-like structures in HNs

Mock-infected and TBEV-infected HNs were examined with transmission electron microscopy at two time points (3 and 12 days p.i.) to delineate TBEV-induced morphological structures that were associated with early and late phases of the infection. Compared to mock-infected HNs, TBEV-infected cells exhibited a broad range (a complex set) of virus-induced subcellular structural changes at both time points.

**Figure 3:**
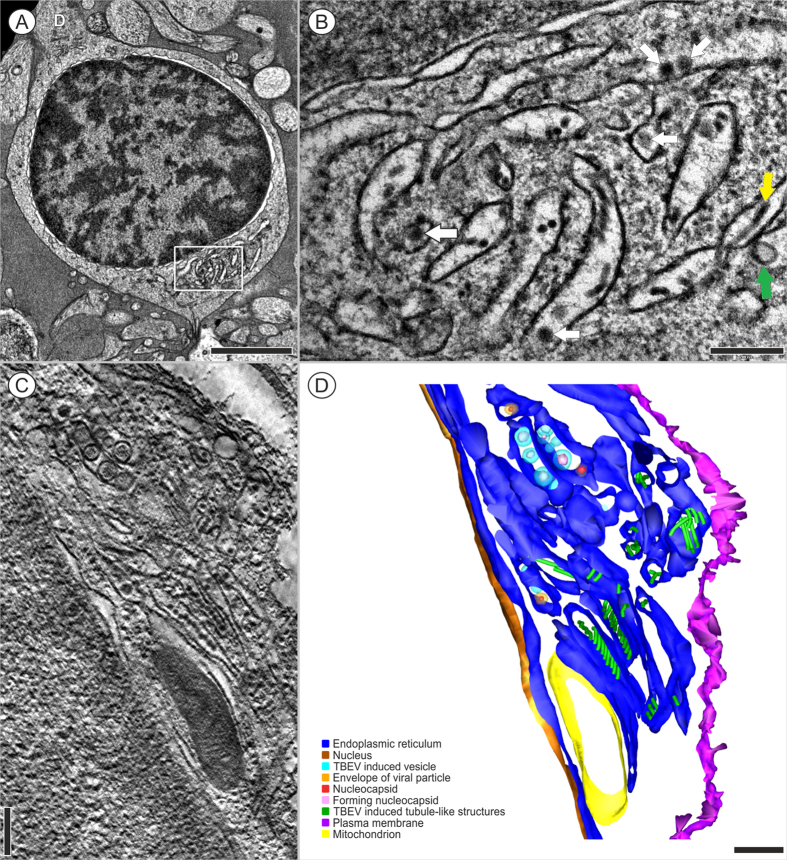
HNs infected with TBEV for 3 days. (**A**) A neuron with two cytoplasmic extensions on opposite sides of the cell body. D- Dendrite. (**B**) Detail of the boxed region in (**A**) shows the RER, which contains viral particles (44.75 nm, n = 4, white arrows), virus-induced vesicles (green arrow), and tubule-like structures (yellow arrow). (**C**,**D**) The presence of tubule-like structures (22 ± 1.3 nm, N = 51) inside the RER. (**C**) A slice of the tomogram was rendered as (**D**) a 3D reconstruction of a single axis of the tomogram. A series of images were collected in a ±65° tilt range with 0.65° increments. Pixel resolution: 1.1 nm. This single-axis tomogram is shown in movie S1. Bars: (**A**) 2 mm, (B-D) 200 nm. The transmission electron microscope images were acquired with (**A**,**B**) a JEOL 1010 80 kV and (**C**,**D**) a JEOL F2100 200 kV.

At 3 days p.i., HN RER cisternae contained several resident virions (approximately 45 nm in diameter), virus-induced vesicles, and tubule-like structures (Fig. 3). Virus-induced vesicles were often arranged in two tightly apposed cisterns of the RER. Some of these vesicles contained electron-dense material, which represented newly-formed nucleocapsids (Fig. 3C,D). In other parts of the RER compartment, the cisterns accommodated tubule-like structures that ran either in parallel lines or in different directions (Fig. 3C,D, movie S1). The tubule-like structures measured 22 ± 1.3 nm (N = 51) in diameter.

**Figure 4:**
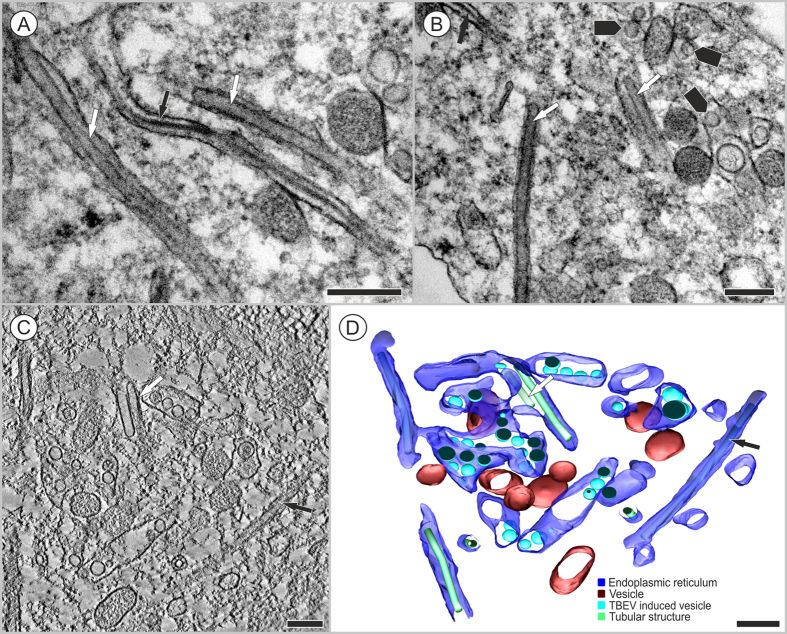
Tubule-like structures of different diameters were localized inside a single neuron infected with TBEV. Transmission electron microscope images were acquired at (**A**) 3 days p.i. or (**B**) 12 days p.i. (**A**) Inside the ER, tubules of 43.8 ± 4.3 nm (N = 7) in diameter (white arrows) were observed; other cisternae contained tubules of 22 ± 1.3 nm in diameter (black arrow). (**B**) The TBEV infection induced ER rearrangements (black arrowheads). (**C**) A slice of the tomogram and (**D**) a 3D reconstruction of a single axis tomogram. Tilt series images were collected in the ±65° tilt range with 1° increments. Pixel resolution: 0.8 nm. This single-axis tomogram is shown in movie S2. Bars: 200 nm. (**A**–**D**) The transmission electron microscope images were acquired with a JEOL F2100 200 kV.

The tubule-like structures were frequently observed at both investigated time points in TBEV-infected HNs. In several cases, tubule-like structures with different diameters (e.g., 22 ± 1.3 nm and 43.8 ± 4.3 nm, N = 7) were observed separately in the RER cisternae of single cells (Fig. 4A–D, movie S2); typically, virus-induced vesicles and virions were observed in neighboring RER spaces (Fig. 4B–D; Fig. 3C,D).

**Figure 5:**
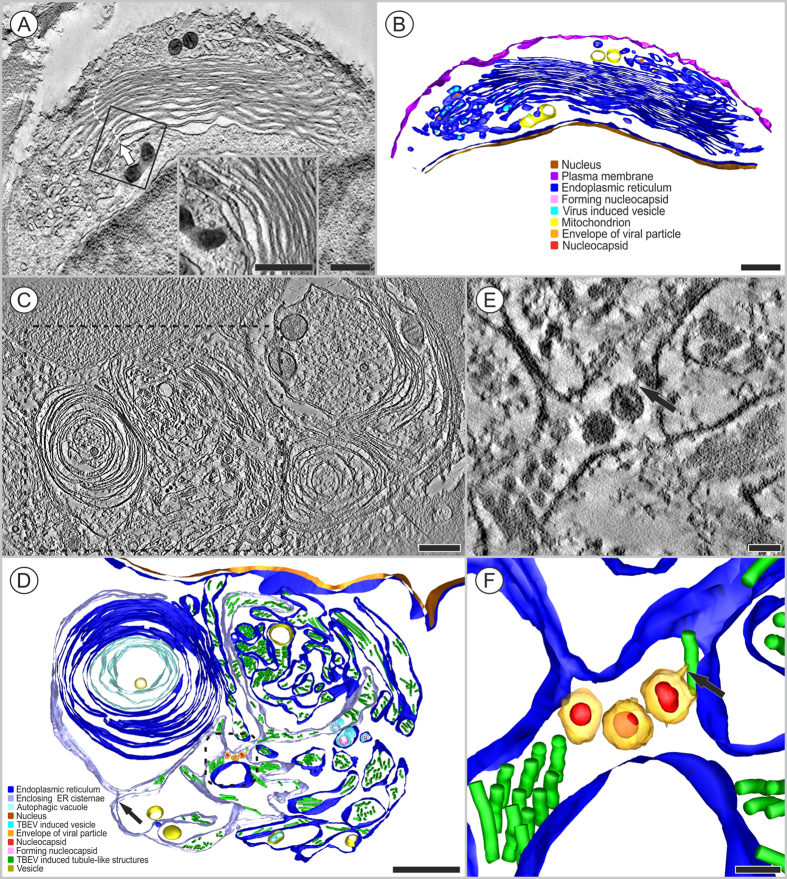
Proliferation of the endoplasmic reticulum observed in HNs infected with TBEV. Transmission electron microscope images were acquired at (**A**,**B**) 3 days p.i. and (**C**–**E**) 12 days p.i. (**A**) TBEV particles and TBEV-induced vesicles are located inside the proliferated and reorganized cisterns of the rough endoplasmic reticulum; the boxed region is enlarged in the inset. (**B**) The image in (**A**) was rendered as a colored 3D model. (**C**) Large whorls are clearly observed in abnormal endoplasmic reticulum. (**D**) The boxed region in (**C**) is rendered in a 3D model to clarify the different components. (**D**) Lamellar whorls are surrounded by cisternae (light purple) arising (arrow) from the rough endoplasmic reticulum (blue), which accommodates tubule-like structures (green). The central part of the whorls comprises concentric circles of flattened ER cisternae (light blue). We observed several lipid droplets (yellow) in proximity of the whorls. (**E**) Detailed image of the boxed region in (**D**) shows the connection between a TBEV particle and a tubule-like structure (arrow) inside the rough endoplasmic reticulum. (**F**) The image in (**E**) was rendered as a colored 3D model. (**A**) A single axis tomogram; ±65° tilt range with 0.65° increments, pixel resolution: 2.2 nm; (**C**) a single axis tomogram, 2 × 2 montage, ±65° tilt range with 1° increments, pixel resolution: 0.8 nm. Bars: (**A**–**D**) 500 nm, (**E**,**F**) 50 nm. (**A**–**F**) The transmission electron microscope images were acquired with JEOL F2100 200 kV.

A prominent morphological change in TBEV-infected HNs was proliferation of the RER membranes that harbored sites of TBEV replication (Fig. 5A,B, movie S3). The proliferative parts of the RER were devoid of ribosomes (Fig. 5A, inset; movie S3, white arrows). At the later time point (12 days p.i.), proliferated RER had rearranged into large whorls (Fig. 5C,D, movie S4). The 3D reconstruction showed complex, lamellar whorls that enclosed a central space containing the cytoplasm of the same structure, and an electron-density as outside, limited by four membranes (Fig. 5D, light blue, movie S5). Numerous flattened ER cisternae were located on the periphery (Fig. 5D, blue). Electron tomography confirmed a continuation/connection of this peripheral part with the RER space, which accommodated TBEV-induced structures (vesicles or tubule-like structures) (Fig. 5D). These observations suggested that the membranous whorls were formed from the peripheral parts of the ER due to extensive ER stress. The large whorls did not contain virions or virus-induced structures. The central part of the whorl had features of an autophagosome, but it did not show signs of degraded content. Finally, we observed a tiny connection between two primary virus-induced structures, as shown in detail in a slice of the tomogram (Fig. 5E) and in the 3D model (Fig. 5F; movie S5).

### Signs of autophagy observed in TBEV-infected HNs

**Figure 6:**
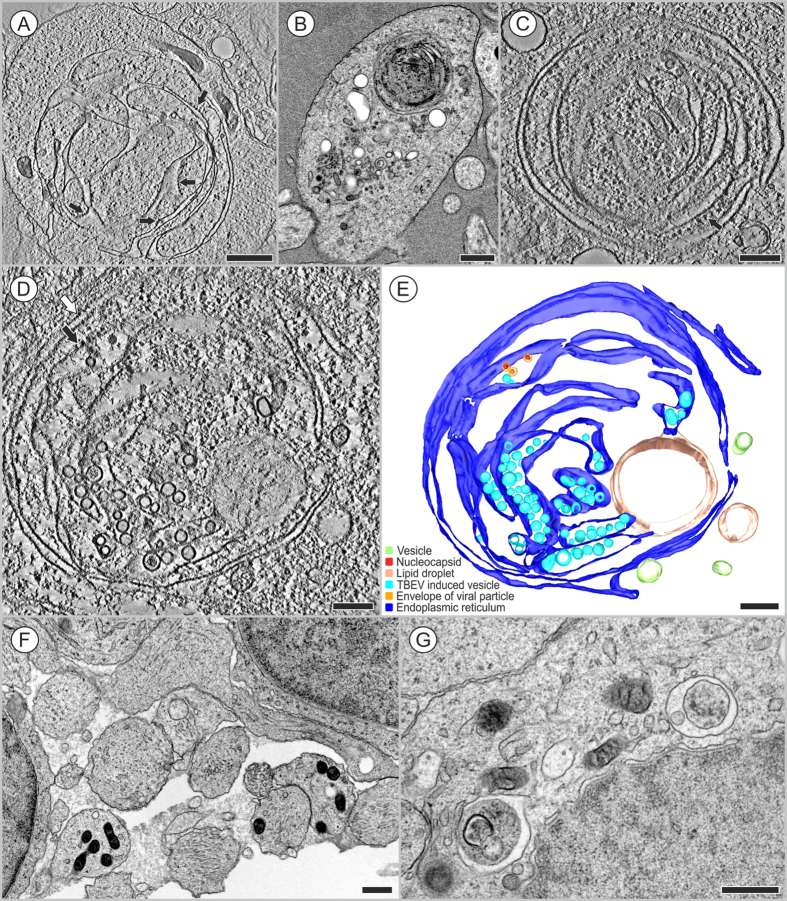
Formation of autophagic vacuoles in HNs infected with TBEV. Transmission electron microscope images were acquired at (**A**–**C**) 3 days p.i., (**D**,**E**) 12 days p.i., and in (**F**,**G**) mock-infected cells. (**A**,**B**) The RER (ribosomes are indicated with black arrows), which contain TBEV particles and virus-induced structures, were nearly completely sequestered by peripheral cisterns (see electron tomography in supplement) in neuronal extensions. (**C**) Detail of (**B**) shows the coiled RER with TBEV-induced structures. (**D**) The cisterns of the RER (ribosomes indicated with black arrow) with replicating TBEV particles and virus-induced vesicles are surrounded by one flattened ER cistern that nearly encloses this space, and a lipid droplet. Enclosing cisternae of the ER were devoid of ribosomes (white arrow). (**E**) 3D model of (**D**). (**F**) Similar vacuoles/autophagosomes were not observed in control neuronal extensions. (**G**) Several vacuoles that sequestered cell parts (debris, fragments of degraded membranes) were found in the cell body of control neurons. Bars: (**A,B**,**F**,**G**) 500 nm, (**C**–**E**) 200 nm. (**A**,**C**,**D**) Slices of a single axis tomogram; ±65° tilt range with 1° increments, pixel resolution: (**A**) 1.1 nm, (**C**) 0.7 nm; (**D**) 0.8 nm. Transmission electron microscope images were acquired with (**A**,**C**–**E**) JEOL f2100 200 kV and (**B**,**F**,**G**) JEOL 1010 80 kV.

**Figure 7:**
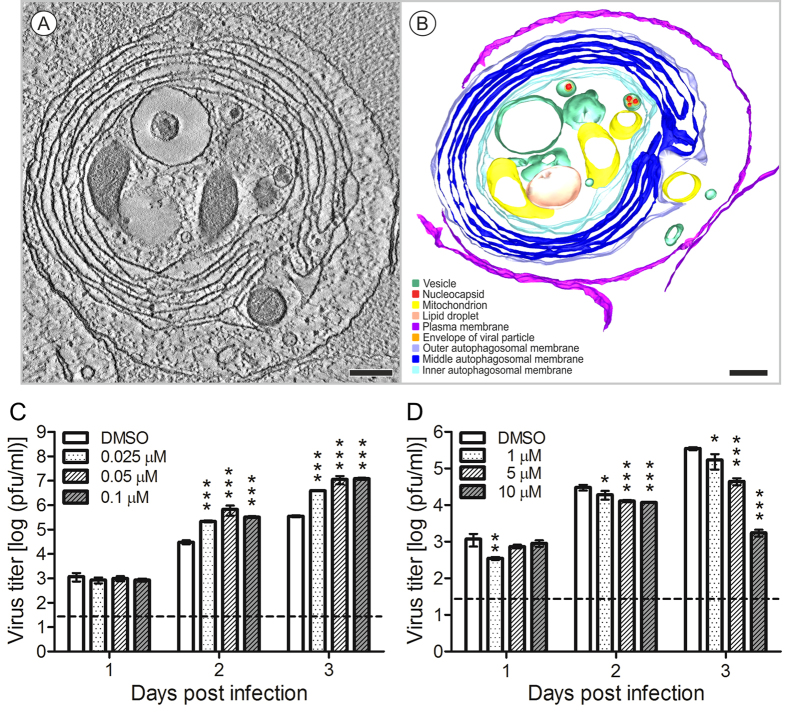
An autophagosome in a neural extension observed 3 days after TBEV infection. The cytoplasm contains mitochondria and TBEV particles in vacuoles, encircled by several double membranous structures, apparently of RER origin (but devoid of ribosomes). (**A**) A slice of a single axis tomogram; ±65° tilt range with 0.6° increments, pixel resolution: 0.8 nm. (**B**) A 3D model of (**A**). Bars: 200 nm. (**A**,**B**) Transmission electron microscope images were acquired with JEOL F2100 200 kV. (**C**,**D**) Autophagy enhanced TBEV production in human neuroblastoma cells. The cells were pretreated with the solvent control (DMSO), rapamycin (0.025, 0.05, or 0.1 μM) (**C**), or spautin-1 (1, 5, or 10 μM) (**D**) then infected with TBEV at m.o.i. of 0.1 pfu per cell for 24, 48, or 72 hours. The culture supernatants were collected for plaque assay on PS cells. The virus titers (pfu/ml) are shown as the means ± SEM. The horizontal dashed line indicates the minimum detectable threshold. ^*^p < 0.05; ^**^p < 0.01; ^***^p < 0.001.

In several cases, particularly in HN extensions, which represented dendrites, we observed that the TBEV replication space was enclosed by flattened ER cisternae. This suggested the formation of autophagosomes that sequestered cell structures damaged by TBEV. Electron tomography was performed to view the complex architecture of these structures. In most cases, the sequestration was incomplete, as shown in Fig. 6A–E (movie S6 and movie S7), even when the projection images suggested that the structure might be interpreted as an autophagic vacuole (Fig. 6B). The TBEV particles and virus-induced vesicles resided in the RER (Fig. 6A,C,D, black arrows), but the enclosing ER cisterns lacked ribosomes (Fig. 6D, white arrows). Lipid droplets were also found associated with these structures (Fig. 6D,E). No morphologically similar structures were found in the extensions of mock-infected HNs (Fig. 6F). However, in the perikaryon (Fig. 6G), several autophagosomes or autophagolysosomes (with signs of degraded material) were observed. In some TBEV-infected HN extensions, we observed autophagosomes with all the typical morphological features (Fig. 7 and movie S8). These autophagosomes were limited by numerous membranes that did not have ribosomes, but they resembled rearranged, small, membranous ER whorls. In the 3D reconstruction, we have identified numerous membranes that sequestered intact mitochondria, lipid droplets, and vacuoles within the inner space with TBEV particles (Fig. 7B).

### Autophagy induction enhances TBEV replication

To study whether autophagy plays a role in TBEV replication, we treated human neuroblastoma cells with rapamycin, an autophagy inducer. The cells were subsequently infected with TBEV at m.o.i. 0.1 pfu per cell. Culture supernatants were harvested after 24, 48, and 72 hours, and viral titers were determined by plaque assay. The viral yields were increased in cells treated with a range of concentrations rapamycin on day 2 and 3 p.i. (Fig. 7C). The induction levels were quite high reaching 1.5 log_10_ pfu/ml higher titers in cells treated either with 0.05 or 0.1 μM of rapamycin in comparison with the untreated control on day 3 p.i. (Fig. 7C).

### Suppression of autophagy reduces TBEV replication

To investigate the effect of spautin-1, a compound known to be an effective autophagy inhibitor, on viral replication in the neuronal cells, we tested its effects on TBEV growth in human neuroblastoma cells. The cells pretreated with a range of concentrations of spautin-1 for 30 min, were infected with TBEV at m.o.i. 0.1 pfu per cell, after which the incubation was continued in the presence of the drug. Culture supernatants were harvested after 24, 48, and 72 hours, and viral titers were determined by plaque assay. Treatment with a range of concentrations of spautin-1 showed a profound, dose-dependent inhibition of the yield of infectious virus, with statistically significant drops in TBEV titer of more than two orders of magnitude at the highest spautin-1 concentration (10 μM) on day 3 p.i. (Fig. 7D).

### Transport of the TBEV in the neuron

**Figure 8:**
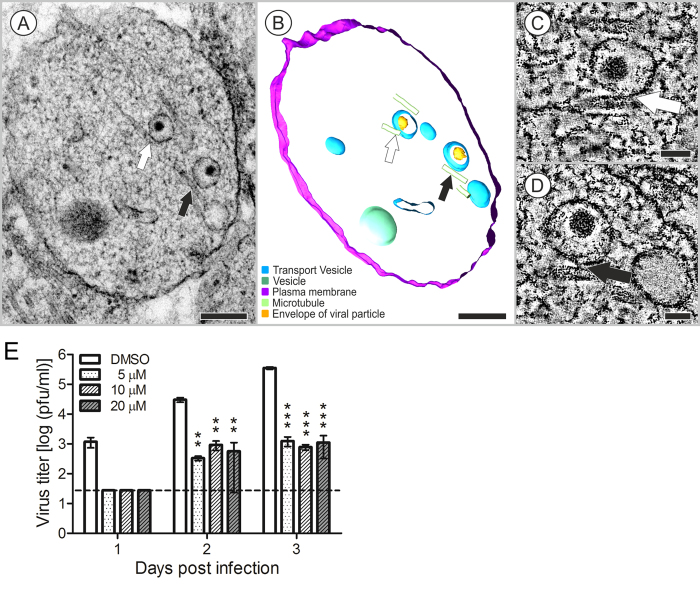
Two vacuoles that accommodated TBEV particles in a neuronal extension at 12 days after infection. (**A**–**D**) Arrows indicate connections between vacuoles and microtubules. (**A**) The projection image and (**B**) the 3D model. (**C**,**D**) Images show slices of a double-axis tomogram acquired with a ± 60° tilt range in 0.6° increments; pixel resolution: 0.55 nm Bars: (**A**,**B**) 200 nm; (**C**,**D**) 50 nm. Transmission electron microscope images were acquired with (**A**) JEOL 1010 80 kV and (**C**,**D**) JEOL F2100 200 kV. (**E**) Nocodazole treatment inhibits TBEV replication in human neuroblastoma cells. The cells were pretreated with the solvent control (DMSO), or nocodazole (5, 10, or 20 μM) then infected with TBEV at m.o.i. of 0.1 pfu per cell for 24, 48, or 72 hours. The culture supernatants were collected for plaque assay on PS cells. The virus titers (pfu/ml) are shown as the means ± SEM. The horizontal dashed line indicates the minimum detectable threshold. ^**^p < 0.01; ^***^p < 0.001.

**Figure 9:**
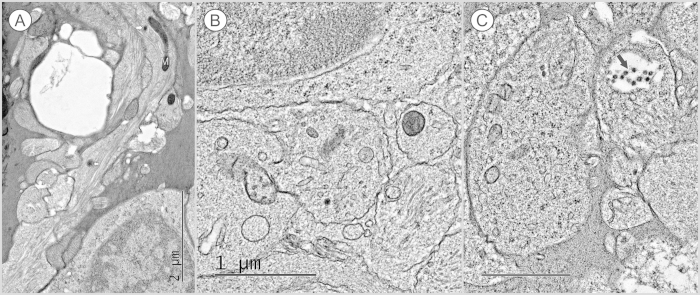
Mock infected HNs examined at 3 days p.i. (**A**,**B**) HN extensions contained a few probable secretion granules with electron-dense cores, with diameters of (**A**) about 70 nm and (**B**) 90 nm, that were in direct contact with the cytoplasm. (**C**) Different vesicles of 43.8 nm (n = 5) in diameter were located outside the cells (black arrow). Bar 1 μm. (**A**–**C**) Transmission electron microscope images were acquired with JEOL 1010 80 kV.

One projection image of a 40-nm thick ultrathin section showed that, in the extensions of TBEV-infected HNs, two virions (48.2 nm and 48.9 nm diameters, with nucleocapsids of 30.3 and 30.9 nm diameters, respectively) were located separately inside vacuoles that were directly connected to cellular microtubules (Fig. 8A). The presence of that connection was confirmed by examining a double-axis electron tomography (±60° tilt range with 0.6° increments, pixel resolution: 0.55 nm), and then creating a 3D reconstruction (Fig. 8B, movie S9). Both connections are shown in detail on slices of the tomogram (Fig. 8C,D, arrows). Extensions of mock-infected HNs contained vesicles with electron-dense cores with diameters that ranged from 70 to 90 nm; those vesicles were observed in direct contact with the cytoplasm (Fig. 9A,B). Electron-dense granules (similar to the vacuoles that contained virions in Fig. 8A) were found in close proximity to bunches of microtubules and mitochondria, as seen in the transverse section (Fig. 9A). Other vesicles, 43.8 nm in diameter, but different in structure from virions (for comparison see Fig 8A), were observed in the control cells (Fig. 9C).

We used nocodazole to assess the effect of microtubule destabilizing agent on virus growth in human neuronal cells. Nocodazole is known to induce depolymerization of actin filaments and microtubules. Human neuroblastoma cells pretreated with a range of non-cytototoxic concentrations of nocodazole for 30 min were infected with TBEV at m.o.i. 0.1 pfu per cell, after which the incubation was continued in the presence of the drug. Culture supernatants were harvested after 24, 48, and 72 hours, and viral titers were determined by plaque assay. Treatment with a range of concentrations of nocodazole showed a profound inhibition of the yield of infectious virus, with statistically significant drops in TBEV titer of more than two orders of magnitude at the all concentrations of nocodazole on days 2 and 3 p.i. (Fig. 8E).

## Discussion

We demonstrated that neuronal TBEV infections produced cell-free, infectious virus by using the supernatant from TBEV-infected HNs to infect PS cells and measuring the viral titers with a plaque assay. TBEV replication was detected in the form of released virions on day 3 p.i., and the culture supernatant maintained a virus titer of approximately 10^7^ pfu/ml until the end of the experiment (Fig. 1A). This finding suggested that the TBEV-infected HNs had apparently transitioned from an acute to a persistent infection. In a previous study, we compared TBEV growth in human neuroblastoma, glioblastoma, medulloblastoma, and PS cells. That study showed that virus replication in neural cell lines was more than 100 times more efficient than in the PS cells^14^. In the present study, we showed that primary HNs were also highly sensitive to TBEV infection, and they also produced high virus titers.

A previous study in primary mouse neurons showed that TBEV antigen accumulated in the dendrites of infected neurons^15^. Viral proteins were synthesized principally in the neuronal cell bodies in the early stages of infection, but they were distributed to dendrites later^15^. In the present study, TBEV-infected HNs also showed antigen accumulation in dendrites (Fig. 1B, arrows), but this was not a frequent event. At early time points after infection, virus antigen was present in practically the whole body of the neuron (Figs. 1B,2); at later time points, antigen accumulation appeared mainly in highly reorganized, proliferated RER (demonstrated in the co-localization experiments with the PDIA3 antigen) (Fig. 2), and only occasionally in dendrites. Viral protein accumulation in dendrites may affect neural function^15^ and TBEV infections can arrest neurite outgrowth^17^. It was hypothesized that viral protein accumulation in dendrites might affect the distribution and function of host proteins, which in turn, might cause neural dysfunction and cellular degeneration^15^.

Our results suggested that vesicles containing TBEV particles were transported in infected neurons, based on observations that TBEV-containing vesicles were associated with microtubules in HNs (Fig. 8A–D). In human neuroblastoma cells, non-cytotoxic concentrations of nocodazole, a compound which disrupts microtubules by binding to β-tubulin and preventing formation of one of the two interchain disulfide linkages and thus inhibiting microtubules dynamics, resulted in significant reduction in virus infectivity (Fig. 8E). Viral spread in neurons is generally mediated by fast axonal transport, a microtubule-associated, anterograde and retrograde transport system. In West Nile virus (WNV) infections, transneuronal viral spread required axonal release of viral particles. WNV underwent bidirectional spread in neurons, and axonal transport promoted viral entry into the CNS^18^.

Here, we also described the morphology and 3D organization of TBEV-induced, tubule-like structures located in the RER of infected HNs (Figs. 3B,4A–D). Similar structures were previously demonstrated in the RER of other vertebrate or arthropod cells infected with TBEV^19^, Langat virus^20^, and mosquito-borne flaviviruses^16^,^21^,^22^. In a previous study, we observed virus-induced vesicles and viral particles directly attached to tubule-like structures in the RER of TBEV-infected human primary astrocytes^19^. The tubule-like structures were only occasionally seen in acutely infected cells, but the number of tubules increased dramatically in persistently infected cells^20^. Unlike previous studies, we frequently observed the presence of tubule-like structures in TBEV-infected neurons. To the best of our knowledge, this study was the first to demonstrate the simultaneous presence of two sizes of tubule-like structures in the RER of single infected cells (Fig. 4A–D). Inside the RER, we observed tubules that were 43.8 ± 4.3 nm (N = 7) in diameter, and in other cisternae, we observed tubules that were 22 ± 1.3 nm in diameter (Fig. 4A–D). Previous studies reported tubule-like structures that ranged from 50 to 100 nm in diameter and from 100 nm to 3.5 μm in length^20^,^21^,^22^,^23^. In TBEV-infected human astrocytes, we previously observed tubule-like structures of 17.9 nm (±0.2 nm) in diameter^19^. The function of tubule-like structures is not clear. These structures may represent features of replication, aberrant structures, or features of a cellular process that aims to restrict the infection^20^. With immunofluorescence and confocal microscopy, we observed fibrillary structures composed of viral E protein in cisterns of the ER; these structures suggested that E protein was present in the tubule-like structures (Figs. 1B,2). The functional contribution of these tubule-like structures to TBEV replication should be addressed in future studies.

Our detailed 3D ultrastructural analysis clearly demonstrated that the TBEV infection triggered a remarkable alteration in the ER membrane structures of HNs (Fig. 5B,D). Previous studies have shown alterations in ER membranes by flaviviruses, which included formations of vesicle pockets and convoluted structures that represented a platform for viral RNA replication and virion assembly^15^,^24^,^25^. These replication compartments also shielded double-stranded RNA from host cell-intrinsic surveillance mechanisms^24^,^25^,^26^.

In TBEV-infected mice, Hirano *et al.*^15^ described characteristic ultrastructural changes in neurite membranes, called LMSs. They hypothesized that LMSs were formed by membrane reconstitutions triggered by the viral replication, and that the LMS might serve as a platform for dendritic viral replication and virion assembly^15^. In the present study, we observed TBEV replication sites in dendrites, but also in perinuclear regions of TBEV-infected HNs. However, our 3D tomography data strongly suggested that, in several cases, the TBEV replication sites were enclosed inside newly formed autophagosomes. Moreover, we observed virions inside autophagosomes that were surrounded by numerous membranes (Figs. 5,6,7) with many typical morphological features. Autophagosome-limiting membranes did not have ribosomes, and they always had two or more limiting membranes^27^. Additionally, we observed interactions with lipid droplets (LDs)^28^ in both newly forming autophagosomes and fully-formed autophagosomes of TBEV-infected HNs. A recent report noted the existence of a physical connection between the endoplasmic reticulum and newly forming autophagosomes^29^,^30^. We confirmed this observation (Fig. 5C,D and Fig. 6).

Upon autophagy induction, the initial event is the formation of a membranous cistern called the phagophore, or isolation membrane. Autophagy begins with the isolation of double-membrane structures in the cytoplasm. Later, these membrane structures elongate and mature. The elongated double-membranes form autophagosomes (large vesicles with diameters of 500–1500 nm in mammalian cells), which become mature with acidification; then, they fuse with lysosomes to become autolysosomes. The sequestered content is then degraded by lysosomal hydrolases^31^.

There are many proposals for the role of autophagy during virus infection in neurons. In post-mitotic, long-lived cell types, such as neurons, basal and stress-induced autophagy may be particularly important for maintaining cellular health. In addition, it represents an important neuronal antiviral defense mechanism for the sequestration and elimination of viral proteins, viral particles, and viral replication complexes^32^. Thus, autophagy represents an adaptive, pro-survival process, rather than a maladaptive, pro-death response, during CNS viral infection^32^. Autophagy has been shown to restrict the replication of several viruses in neurons, including Rift Valley fever virus^33^, Sindbis virus^34^, and herpes simplex virus type 1^35^. However, the growth of WNV has been shown to be independent of WNV-induced autophagy in neuronal cultures^36^. Although autophagy is a cellular homeostatic mechanism involved in the antiviral response, it can be also subverted to support viral growth. Virus-induced autophagy promoted the replication of dengue virus^37^,^38^, enterovirus 71^31^, poliovirus, and coxsackievirus B4^39^. Japanese encephalitis virus growth is dependent on autophagy activation during the early stages of infection^40^. Our study was the first to demonstrate that autophagy occurred during TBEV replication in HNs, and is also the first report that autophagy enhances TBEV replication. We treated TBEV-infected neuroblastoma cells with rapamycin (inducer of autophagy, as inhibition of mTOR mimics cellular starvation by blocking signals required for cell growth and proliferation), and spautin-1 (highly specific and potent autophagy inhibitor in mammalian cells, which promotes the degradation of Vps34 PI3 kinase complexes by inhibiting two ubiquitin specific peptidases, USP10 and USP13 that target the Beclin1 subunit of Vps34 complexes) and investigated the effect of the treatment of TBEV growth. The induction of autophagy in human neuroblastoma cells by rapamycin increased TBEV replication (Fig. 7C), whereas the inhibition of autophagy by spautin-1 reduced significantly viral titers (Fig. 7D), indicating that autophagy positively regulates TBEV replication.

As mentioned above, we observed direct interactions between autophagosomes and LDs in TBEV-infected HNs (Fig. 6D,E, Fig. 7). A previous report showed that autophagosomes could target cellular lipid stores (LDs) to generate energy for the cell^41^. For example, dengue viral-infection-induced autophagy stimulated the delivery of lipids to lysosomal compartments, which resulted in the release of free fatty acids. These fatty acids underwent β-oxidation in the mitochondria to generate ATP, which produced a metabolically favorable environment for viral replication^28^. Thus, aside from the many roles of autophagy in regulating cellular homeostasis, its regulation of lipid metabolism can represent a major contributor to robust viral replication^41^. The role of autophagy as a provider of free fatty acids as an energy source for RNA replication during viral infection will be a subject of our further studies.

In conclusion, we directly investigated the morphological changes induced by TBEV infections in HNs with advanced high-pressure freezing, freeze-substitution, and electron tomography techniques, with the purpose of achieving optimal preservation of ultrastructure for electron tomography visualization of cellular architecture. These methods enabled clear visualization of connections between microtubules and vacuoles that harbored TBEV virions in neuronal extensions, connections between tubule-like structures and virions, 3D organizations of proliferating endoplasmic reticulum membranes, membranous whorls, and the formation of autophagic vacuoles. These data provided insight into the process of TBEV-induced injury in HNs, and our findings will promote future studies that aim to understand the molecular mechanism of TBEV infection in the human CNS.

## Methods

### Virus and cells

The virus used in this study was the TBEV strain, Neudoerfl, kindly provided by Professor F. X. Heinz from the Medical University in Vienna. The strain was originally isolated from the tick *Ixodes ricinus* in Austria in 1971. The virus represents a prototype strain of the European subtype of TBEV and was characterized extensively, including its genome sequence and the 3D structure of its envelope protein E^42^. The virus underwent four passages in the brains of suckling mice before use in this study.

Human neurons (HNs) were isolated from the human brain and characterized by immunofluorescence with antibodies specific to neurofilament, microtubule associated protein 2 (MAP2), and β-tubulin III (purchased at passage zero from ScienCell Research Laboratories, Carlsbad, CA). HNs were propagated in Neuronal Medium (ScienCell) with 1% neuronal growth supplement and 1% penicillin/streptomycin (ScienCell) at 37 °C and 5% CO_2_. In all experiments, cells were used at passage zero.

Human neuroblastoma cells UKF-NB-4^14^ were cultured at 37 °C and 5% CO_2_ in Iscove’s modified Dulbecco’s medium (IMDM) supplemented with 10% fetal bovine serum (Sigma) and 1% mixture of penicillin and streptomycin (Sigma).

Porcine kidney stable (PS) cells^43^ were cultured in L-15 medium supplemented with 3% newborn calf serum and 1% penicillin/streptomycin (Sigma-Aldrich) at 37 °C.

### Viral growth in HNs

HNs were seeded onto gelatin (0.2%) coated wells of 96-well plates at 20,000–25,000 cells/cm^2^. After establishing the culture, cells were inoculated with the virus diluted in culture medium to 5 multiplicity of infection units (MOI). Virus-mediated cytopathic effect (CPE) was investigated with light microscopy. At 0, 3, 5, 7, and 12 days p.i., supernatant medium from appropriate wells was collected and frozen at −70 °C. Viral titers were determined by plaque assay.

### Plaque assay

PS cells were used to determine virus titers according to a protocol described previously, with minor modifications^44^. Tenfold dilutions of the virus samples were placed in 24-well tissue culture plates, and suspended PS cells were added (5×10^4^ cells per well). After incubating for 4 h, the suspension was overlaid with carboxymethylcellulose (1.5% in L-15 medium). After incubating for 5 days at 37 °C, the plates were washed with PBS, and the cells were stained with naphthalene black (Sigma Aldrich). Virus titer was expressed in units of pfu/ml.

### Immunofluorescence staining

TBEV-infected and control HNs were grown on slides. Then, cells were fixed in 4% formaldehyde for 1 h at room temperature, rinsed three times in 0.1 M phosphate buffer (PB) with 0.02 M glycine, permeabilized with 0.1% Triton X-100 with 1% normal goat serum in 0.1 M PB for 30 min, and blocked with 5% normal goat serum. Cells were labeled with flavivirus-specific mouse mAb (1:250; Millipore) for 1.5 h at 37 °C. Flavivirus-specific mAb and rabbit anti-PDIA3 antibody (1:250, Sigma-Aldrich) were used for double labeling. After washing with ten–fold diluted blocking solution, the cells were labeled with goat anti-mouse secondary antibody conjugated with FITC (1:500, Sigma-Aldrich) or goat anti-rabbit secondary antibody conjugated with Atto 550 NHS (1:500, Sigma-Aldrich) for 1.5 h at 37 °C. The cells were counterstained with DAPI (1 μg/ml, Sigma-Aldrich) for 10 min at 37 °C, mounted in 2.5% 1,4-diazabicyclo(2.2.2)octane (Sigma-Aldrich), and examined with an Olympus BX-51 fluorescence microscope equipped with an Olympus DP-70 CCD camera. Confocal microscopy was performed with an Olympus FV-1000; serial Z-series images were acquired in blue, red, and green channels.

### Transmission electron microscopy and electron tomography

TBEV-infected and control HNs were grown on sapphire discs. At either 3 or 12 days p.i., cells were high-pressure frozen in the presence of 20% BSA diluted in Neuronal Medium with a Leica EM PACT2 high-pressure freezer. Freeze substitution was carried out in 2% osmium tetroxide diluted in 100% acetone, as described previously^19^, with a Leica EM AFS2 at −90 °C for 16 h. The samples were than warmed at a rate of 5 °C/h, incubated at −20 °C for 14 h, and finally warmed again at the same rate to a final temperature of 4 °C. The samples were rinsed three times in anhydrous acetone at room temperature and infiltrated stepwise in acetone mixed with SPI-pon resin (SPI) (acetone:SPI ratios of 2:1, 1:1, 1:2, for 1 h at each step). The samples in pure resin were polymerized at 60 °C for 48 h.

Sections were prepared with a Leica Ultracut UCT microtome and collected on 300 mesh cooper grids. Staining was performed with alcoholic uranic acetate for 30 min, and then, lead citrate for 20 min. Images were obtained with a JEOL 2100F or JEOL 1010 transmission electron microscope. For electron tomography, protein A-conjugated 10 nm gold nanoparticles (Aurion) were added to both sides of each section as fiducial markers.

Tilt series images were collected in the range of ±60° to 65°, with 0.6° to 1° increments. Images were acquired with a 200 kV JEOL 2100F transmission electron microscope equipped with a high-tilt stage and a Gatan camera (Orius SC 1000) and controlled with SerialEM automated acquisition software^45^. Images were aligned based on the fiducial markers. Electron tomograms were reconstructed with the IMOD software package. Manual masking of the area of interest was employed to generate 3D surface models^46^.

### Autophagy stimulation and inhibition, and microtubule disruption

Stocks of rapamycin (Sigma-Aldrich) as an autophagy stimulator, spautin-1 (Sigma-Aldrich), an autophagy inhibitor, and nocodazole (Sigma-Aldrich) as a microtubule disruptor, were prepared in DMSO. Monolayers of human neuroblastoma cells in 96-well plates were pretreated with different concentrations of either drug (or DMSO as a negative control) for 30 min at 37 °C and infected with TBEV at a multiplicity of infection of 0.1 pfu per cell. Infection was always performed in triplicate. Supernatants were harvested at 24, 48, and 72 hours p.i. and titers were determined by plaque assay as described above.

### Statistical analysis

Statistical analyses were performed using version 5.04 of the GraphPad Prism5 (GraphPad Software, Inc., USA). Data were transformed by use of the X’ = log(X) formula and analyzed using one-way ANOVA (Dunnett’s Multiple Comparison Test). p-values < 0.05 were considered significant.

## Additional Information

**How to cite this article**: Bílý, T. *et al*. Electron tomography analysis of tick-borne encephalitis virus infection in human neurons. *Sci. Rep.* **5**, 10745; doi: 10.1038/srep10745 (2015).

## Supplementary Material

Supplementary Information

Supplementary Movie S1

Supplementary Movie S2

Supplementary Movie S3

Supplementary Movie S4

Supplementary Movie S5

Supplementary Movie S6

Supplementary Movie S7

Supplementary Movie S8

Supplementary Movie S9
