# Supplementary material for: Electron Tomography Analysis of Tick-Borne Encephalitis Virus Infection in Human Neurons
Source: Sci Rep. 2015 Jun 15;5:10745. doi: 10.1038/srep10745 (PMC4466586; doi:10.1038/srep10745)
Supplement: Supplementary Information [file srep10745-s1.pdf]

Electron tomography analysis of tick-borne encephalitis virus infection in human neurons

Tomáš Bílý, Martin Palus, Luděk Eyer, Jana Elsterová, Marie Vancová, and Daniel Růžek

SUPPLEMENTARY INFORMATION:

Movie S1: The movie shows a Z walk through the tomographic reconstruction of tubule-like structures present inside rough endoplasmic reticulum (corresponding to Figure 3C and D). The single axis tomogram was reconstructed from a ~88.6-nm thick section and divided into 81 slices.

Movie S2: The movie shows a Z walk through the tomographic reconstruction of tubule-like structures of different diameters localized in the rough endoplasmic reticulum of a single neuron (corresponding to Figure 4). A colored 3D model is overlaid on the image, and rotated. This single axis tomogram was reconstructed from a ~46-nm thick section and divided into 56 slices.

Movie S3: The movie shows a Z walk through a proliferating rough endoplasmic reticulum (corresponding to Figure 5A and 5B). It focuses on the details of ER membranes with ribosomes (white arrows) and cisterns devoid of ribosomes. A colored 3D model is overlaid on the image, and rotated. This single axis tomogram was reconstructed from a ~100-nm thick section and divided into 46 slices.

Movie S4: The movie shows a Z walk through the large whorls formed from abnormal endoplasmic reticulum (corresponding to Figure 5C through 5F). A colored 3D model is overlaid on the image, and rotated. Different features are brought into focus. This 2 by 2 montage of a single axis tomogram was reconstructed from a ~40-nm thick section and divided into 46 slices.

Movie S5: The movie shows a Z walk through the formation of autophagic vacuoles (corresponding to Figure 6A). Rough endoplasmic reticulum containing TBEVs and virus-induced structures were nearly completely sequestered by peripheral cisterns. This single axis tomogram was reconstructed from a ~45-nm thick section and divided into 41 slices.

Movie S6: The movie shows a Z walk through the formation of autophagic vacuoles (corresponding to Figure 6C). Detail shows the coiled RER with ribosomes and TBEV-induced structures. This single axis tomogram was reconstructed from a ~41-nm thick section and divided into 61 slices.

Movie S7: The movie shows Z walk through the formation of autophagic vacuoles (corresponding to Figure 6D and 6E). Detail shows the rough endoplasmic reticulum, which included TBEVs. A colored 3D model is overlaid on the image, and rotated. This single axis tomogram was reconstructed from a ~42-nm thick section and divided into 51 slices.

Movie S8: The movie shows a Z walk through an autophagosome in a neuronal extension (corresponding to Figure 7). A colored 3D model is overlaid on the image, and rotated. This single axis tomogram was reconstructed from a ~42-nm thick section and divided into 51 slices.

Movie S9: The movie shows a Z walk through two vacuoles that accommodate TBEV particles in neuronal extensions (corresponding to Figure 8). Detail shows the connection between vacuoles and microtubules. A colored 3D model is overlaid on the image, and rotated. This dual axis tomogram was reconstructed from a ~40-nm thick section and divided into 71 slices.
